# Rab7 GTPase controls lipid metabolic signaling in myeloid-derived suppressor cells

**DOI:** 10.18632/oncotarget.16280

**Published:** 2017-03-16

**Authors:** Xinchun Ding, Wenjing Zhang, Ting Zhao, Cong Yan, Hong Du

**Affiliations:** ^1^ Department of Pathology and Laboratory Medicine, Indiana University School of Medicine, Indianapolis, IN, USA; ^2^ IU Simon Cancer Center, Indiana University School of Medicine, Indianapolis, IN, USA

**Keywords:** Rab7 GTPase, myeloid-derived suppressor cells, lipid metabolism, tumor growth

## Abstract

Lysosomal acid lipase (LAL) is a critical neutral lipid metabolic enzyme that regulates metabolic reprogramming in myeloid-derived suppressor cells (MDSCs) through over-activation of mammalian target of rapamycin (mTOR). Affymetrix GeneChip microarray analysis of MDSCs from LAL deficient mouse (*lal*^−/−^) revealed upregulation of Rab7 GTPase protein, which belongs to a superfamily of small-molecular-weight GTPase known to regulate intracellular membrane trafficking from early to late endosomes and lysosomes. Here, the physical protein-protein interaction between Rab7 GTPase and mTOR has been detected by co-immunoprecipitation in the cell extract of wild type HD1A and *lal*^−/−^ MDSC-like HD1B myeloid cell lines. The GST pull down assay using the recombinant GST-Rab7 GTPase fusion protein showed that Rab7 GTPase interacts with the mTOR N-terminal heat repeat domain. Rab7 GTPase siRNA knocking down reversed the altered lysosome/mTOR distribution and expression levels in HD1B cells. Rab7 GTPase siRNA knocking down in isolated bone marrow *lal*^−/−^ MDSCs or HD1B cells not only reduced over-activation of mTOR and its downstream effector S6, but also decreased glucose consumption, decreased ROS over-production, and increased healthy mitochondria by membrane potential measurement. Inhibition of Rab7 GTPase led to reduced *lal*^−/−^ MDSCs differentiation from bone marrow Lin^−^ progenitor cells, reduced *lal*^−/−^ MDSCs trans-endothelial migration, and reversed *lal*^−/−^ MDSCs suppression of T cell proliferation. Furthermore, inhibition of Rab7 GTPase reduced *lal*^−/−^ MDSCs ability to stimulate tumor cell proliferation *in vitro*, tumor growth *in vivo*, and tumor invasion. Together, these results showed that Rab7 GTPase is critically involved in MDSCs homeostasis and pathogenic functions.

## INTRODUCTION

Sporadic tumor cells arise from various genetic or epigenetic defects. The immune surveillance system in the body detects and destroys newly formed tumor cells, and prevents them from growing and spreading. When immune surveillance is compromised, unchecked tumor cells proliferate and grow freely to form tumor [1]. Inflammatory responses play decisive roles at different stages of tumor development, including initiation, promotion, malignant conversion, invasion, and metastasis [2, 3]. One of manifestations of inflammation is the expansion of myeloid-derived suppressor cells (MDSCs) which are commonly defined by the markers CD11b (integrin α-M) and Gr-1 (Ly6-C/G) in mouse [4]. The hallmark of MDSCs is their ability to suppress immune surveillance (T cells, NK cells etc). Recently, we have made a major breakthrough and identified that MDSCs are able to directly stimulate tumor cell proliferation *in vitro*, and tumor growth and invasion *in vivo* [5–8].

As a control center of signaling for metabolic reprogramming, lysosome plays a vital role in various cellular functions [9]. We have shown that lipid metablic signaling controlled by lysosomal acid lipase (LAL) in lysosome plays a critical role in malformation and malfunction of MDSCs [3]. Fatty acids supply energy, as mitochondrial fatty acid oxidation produces more than twice as much ATP per mole as oxidation of glucose or amino acids. In the absence of LAL that hydrolyzes cholesteryl esters and triglycerides to generate free cholesterol and free fatty acids in the lysosomes, LAL deficient (*lal^−/−^*) MDSCs have to use alternative metabolic pathways to compensate the energy deficit, which leads to increased glycolytic metabolism, increased ATP production, ROS over-production, and impairment of mitochondrial membrane potential as a result of mammalian target of rapamycin (mTOR) over-activation [7, 10]. Ingenuity Pathway Analysis of Affymetrix GeneChip microarray revealed up-regulation of multiple genes in the mTOR signaling pathway [10]. mTOR serves as a nutrient/energy/redox sensor for cell growth and belongs to the phosphoinositide 3-kinase (PI3K)-related protein kinases (PIKK) family [11–13]. In an array of studies recently conducted in our laboratories, we have clearly demonstrated that mTOR critically regulates multi-aspects of *lal^−/−^* MDSCs, including development, systemic expansion, trans-endothelial migration, immune suppression, and direct stimulation of tumor cell proliferation [3, 5–7, 14, 15].

Evidence suggests that membrane trafficking causes mTOR to shuttle to lysosomes and regulate mTOR signaling [16, 17]. The lysosomal membrane acts as a platform for the mTOR signaling. Since LAL is a lysosomal enzyme, lacking the LAL activity influences endomembrane trafficking and changes the mTOR activity. In searching for lysosomal proteins that might control mTOR trafficking and activity, Rab7 GTPases was up-regulated in *lal^−/−^* MDSCs [10]. Through the interaction with its partners, Rab7 GTPase participates in multiple regulatory mechanisms in endosomal sorting, biogenesis of lysosome and phagocytosis [18]. Recently, the specific role of Rab7 GTPase in cancer cell proliferation and invasion begins to unravel. In the literature, Rab7 GTPase is pro-tumorigenic in both aspects [19–21]. However, its role in tumor-promoting MDSCs has never been explored. Here, we identified that Rab7 GTPase regulates the mTOR activity through a direct physical interaction in normal myeloid cells and *lal^−/−^* MDSCs. Inhibition of Rab7 GTPase over-activation reduced various pathogenic functions of *lal^−/−^* MDSCs.

## RESULTS

### Rab7 GTPase interacts with the mTOR complex to influence its downstream signaling

**Figure 1:**
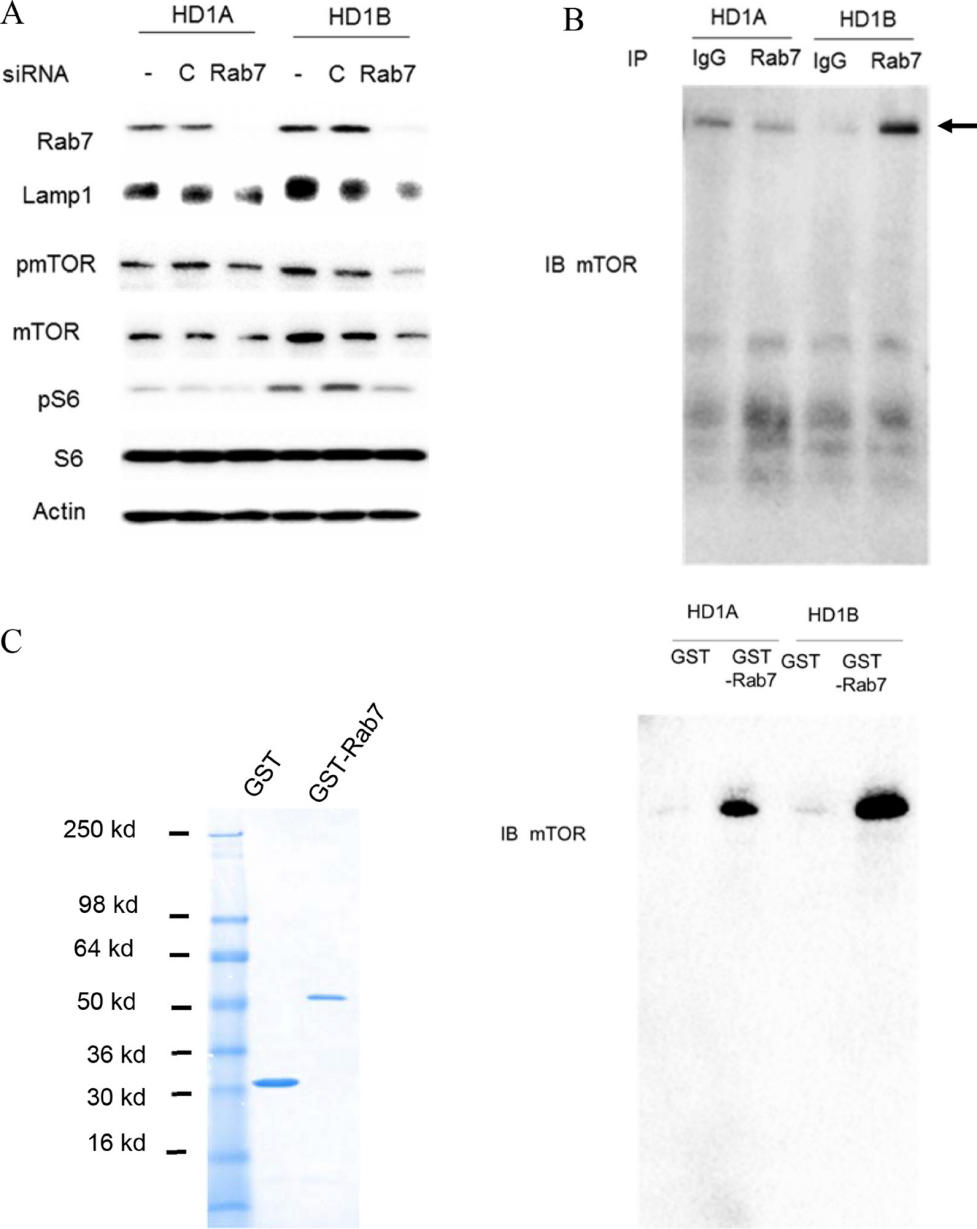
Rab7 GTPase and mTOR interaction (**A**) Knocking down Rab7 GTPase by siRNA reduced LAMP1, mTOR, phosphorylated mTOR and phosphorylated S6 protein levels in HD1B cells. HD1A and HD1B cells were transfected with or without control or Rab7 GTPase siRNA for 3 d, and cell lysates were subjected to Western blot analysis; (**B**) Immuno-precipitation assay of Rab7 GTPase and mTOR interaction. HD1A and HD1B cell lysates were immuno-precipitated by control IgG (IgG) or anti-Rab7 GTPase antibody (Rab7), and detected by anti-mTOR antibody in Western blot analysis; (**C**) GST pulldown assay of Rab7 GTPase and mTOR interaction. Left panel, GST and GST-Rab7 fusion protein were expressed in E.coli and purified by GST column. The purity of recombinant proteins were visualized by coomassie blue staining on SDS-PAGE. Right panel, HD1A and HD1B cell lysates were incubated with purified recombinant GST or GST-Rab7 fusion protein and pull down with GST-beads, and detected by anti-mTOR antibody in Western blot analysis. For A-C, representative results are shown from three independent experiments.

Since both over-activation of the mTOR signaling pathway and increased Rab7 GTPase expression co-exist in *lal^−/−^* MDSCs [10], we hypothesized that the mTOR signaling pathway is regulated by Rab7 GTPase. The Rab7 GTPase was blocked by siRNA transfection in MDSCs-like HD1B cells (*lal^−/−^*) and control wild type HD1A cells [7]. As shown in Figure 1A, HD1B cells had higher expression levels of Lysosomal associate membrane protein 1 LAMP1 and mTOR, but not S6. The Rab7 GTPase siRNA knocking down effectively reduced the Rab7 GTPase protein level in both HD1A and HD1B cells. Interestingly, Rab7 GTPase knocking down reduced expression of LAMP1 in both HD1A and HD1B cells. Rab7 GTPase knocking down also reduced mTOR, pmTOR, and pS6 levels in HD1B cells. These results showed that Rab7 GTPase is critical for activation of mTOR signaling and lysosome genesis.

Based on these observations, immunoprecipitation was performed. As shown in Figure 1B, the intensity of mTOR protein was much stronger in the cell lysate of HD1B cells than that of HD1A cells after immunoprecipitation by anti-Rab7 GTPase antibody. To further confirm this finding, a GST pulldown study was performed using purified recombinant proteins (Figure 1C, left). After incubation with cell extracts of HD1A and HD1B cells, the GST-Rab7 GTPase fusion protein pulled down the intracellular mTOR complex in both HD1A and HD1B cells (Figure 1C, right), with much stronger intensity of mTOR in HD1B cells than that in HD1A cells. As a control, GST pulldown showed a very weak non-specific band in both cell lines. Therefore, Rab7 GTPase is physically associated with mTOR and LAL deficiency leads to the enhanced interaction between these two proteins in myeloid cells.

### Define the mTOR domains interacting with Rab7 GTPase

**Figure 2:**
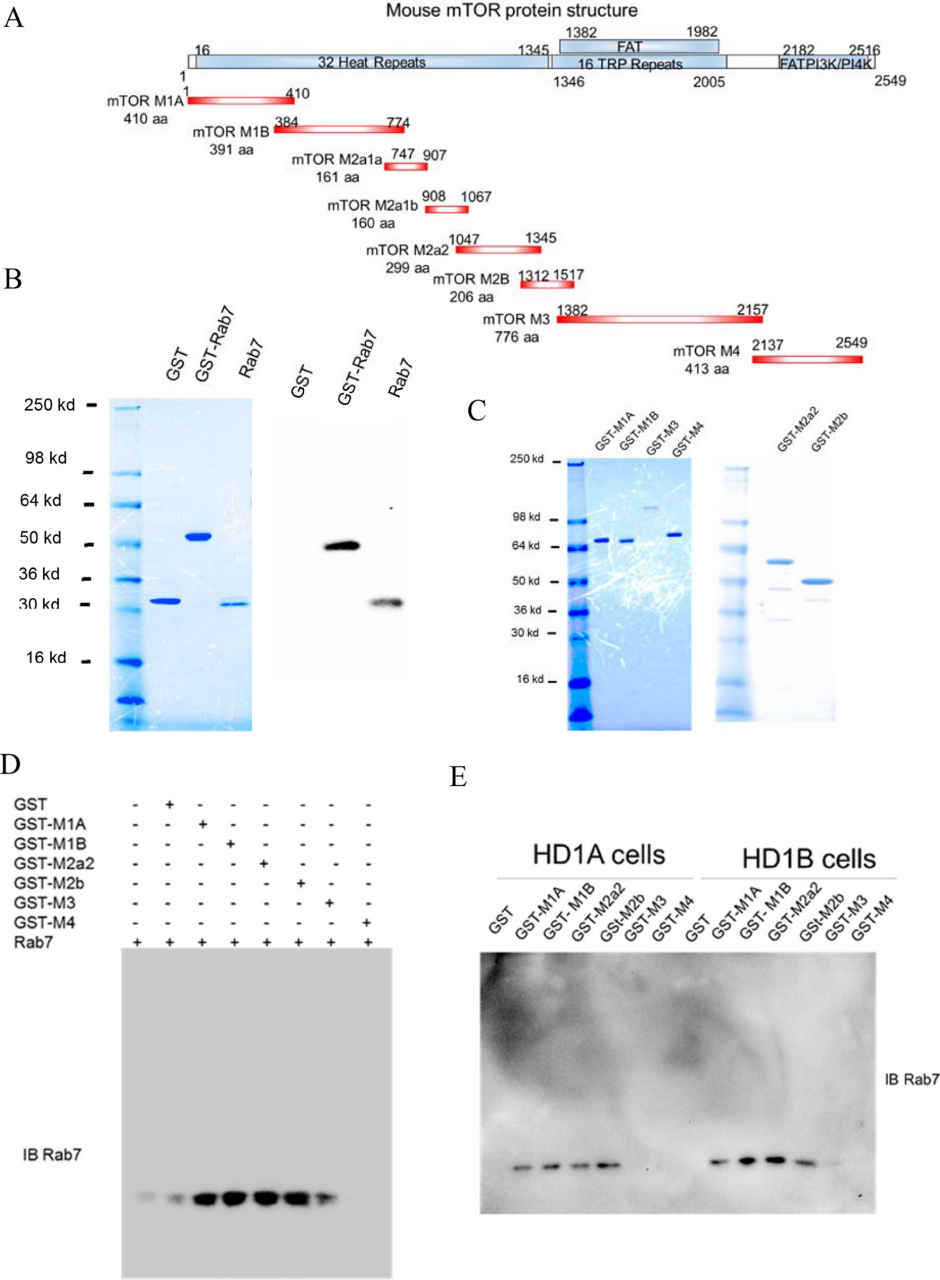
GST pulldown of Rab7 GTPase with mTOR domains (**A**) The schematic diagram of the mTOR domain structure (Upper), and PCR-generated GST-mTOR fusion fragments (Lower); (**B**) GST cleavage of GST-Rab7 fusion protein by thrombin and analyzed by coomassie blue staining (Left), and confirmed by Western blot analysis using anti-Rab 7 GTPase antibody (Right); (**C**) Expression and purification of various recombinant GST-mTOR fragments; (**D**) Pull-down of recombinant Rab7 GTPase by recombinant GST-mTOR fragments; (**E**) Pull-down of endogenous Rab7 GTPase in HD1A or HD1B cells by recombinant GST-mTOR fragments. From B-E, results are representative of three independent experiments.

The mTOR is a very large protein (2516 aa) and contains multiple functional domains (Figure 2A). While it was too difficult to amplify the full length of mTOR, various mTOR fragments were amplified by PCR and subcloned into the pGEX-4T-1 vector (Figure 2A, M1A, M1B, M2a2, M2b, and M3, and M4) to cover the entire mTOR region, and expressed as GST-fusion proteins (Figure 2C). Rab7 GTPase was generated by thrombin cleavage of bacterial expressed GST-Rab7 GTPase fusion protein and used for pulldown assay (Figure 2B). GST was used as a baseline control. As demonstrated in Figure 2D, mTOR fragments of GST-M1A, GST-M1B, GST-M2A2, GST-M2b were able to pull down the recombinant Rab7 GTPase protein, whereas GST-M3 and GST-M4 were not. The same observation was confirmed by GST pulldown of endogenous Rab7 in HD1A and HD1B cell extracts by the recombinant GST-mTOR fragments (Figure 2E). These results indicate that Rab7 GTPase directly interacts with mTOR through the N-terminal heat repeat domain of mTOR.

### Rab7 GTPase controls lysosome cellular distribution in LAL deficient cells

**Figure 3:**
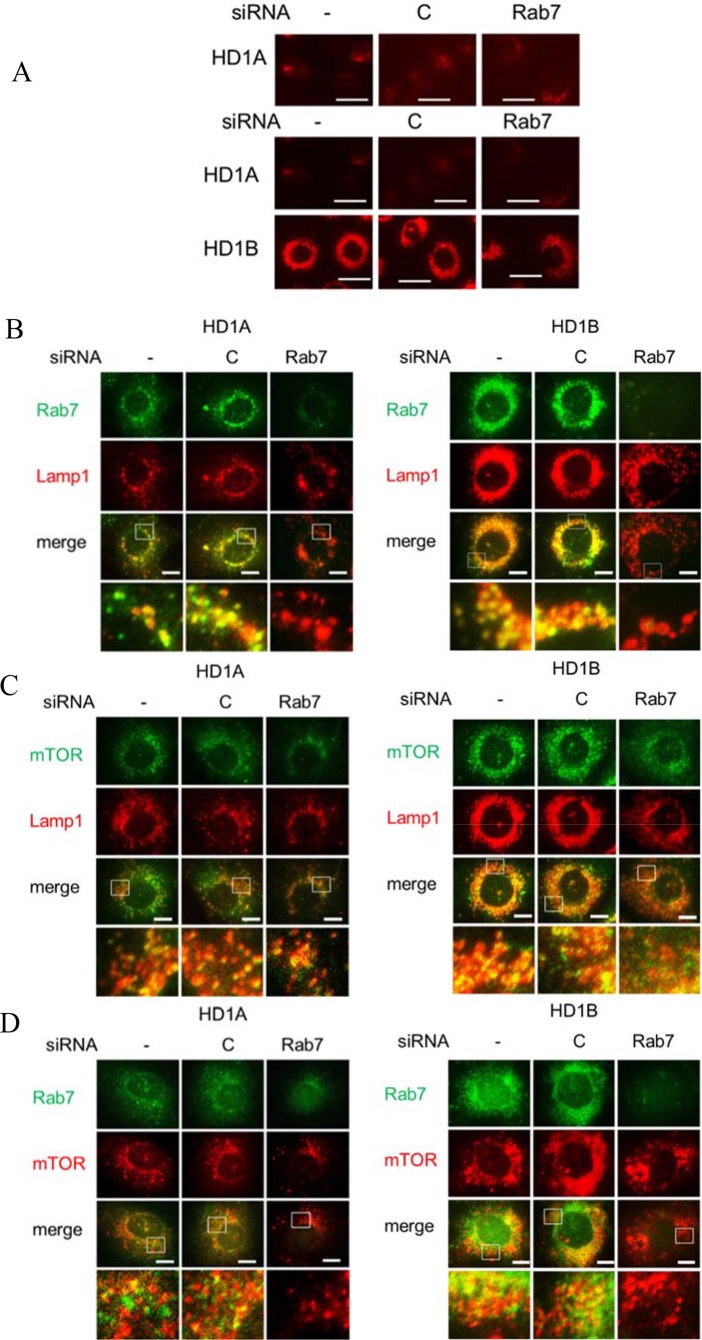
Rab7 GTPase controls lysosome genesis (**A**) LysoTracker Red staining of HD1A or HD1B cells with control or Rab7 GTPase siRNA treatment for 3 d. Lysosomal perinuclear location was intensified in HD1B cells. Top, A longer exposure of HD1A cell; scale bar, 20 μm. (**B**) Co-localization of Rab7 GTPase and LAMP1 by immunofluorescent staining in HD1A and HD1B cells with control or Rab7 GTPase siRNA transfection for 3 d; (**C**) Co-localization of mTOR and LAMP1 by immunofluorescent staining in HD1A and HD1B cells with control or Rab7 GTPase siRNA transfection for 3 d. Notice the mTOR staining intensity was reduced by Rab7 GTPase siRNA knocking down; (**D**) Co-localization of Rab7 GTPase and mTOR by immunofluorescent staining in HD1A and HD1B cells with control or Rab7 GTPase siRNA transfection for 3 d. The mTOR staining intensity was reduced by Rab7 GTPase siRNA knocking down. From A–D, results are representative of five independent experiments. B-D, scale bar, 10 μm. -, no transfection; C, transfected with control siRNA; Rab7, transfected with Rab7 siRNA.

As demonstrated in Figure 3A, LAL deficient HD1B cells significantly induced lysosomal genesis and showed much more LysoTracker positive lysosomes around the perinuclear region than those in wild type HD1A cells. Knocking down Rab7 GTPase by siRNA reduced lysosomal genesis and re-located lysosomes by immunofluorescence co-staining of lysosomal marker LAMP1 and Rab7 GTPase, in which Rab7 GTPase was co-localized with LAMP1 on the lysosomes (Figure 3B and Supplementary Figure 1A). Figure 3B and Supplementary Figure 1A also showed that Rab7 GTPase siRNA effectively knocked down Rab7 GTPase expression in both HD1A and HD1B cells. Similarly, mTOR and LAMP1 were co-localized in HD1B cells and only partially overlapped in HD1A cells (Supplementary Figure 1B). The image confirmed that knocking down Rab7 GTPase reduced lysosomal genesis and re-located lysosomes as evidenced by mTOR and LAMP1 co-localization in HD1B cells (Figure 3C, Supplementary Figure 1B). Since Rab7 GTPase and mTOR are physically interactive, it is important to determine if they are co-localized in HD1A and HD1B cells. In both HD1A and HD1B cells Rab7 GTPase and mTOR were co-localized in a large extent (Figure 3D, Supplementary Figure 1C). Knocking down Rab7 GTPase reduced mTOR signals in both cells (Figure 3D). Therefore, LAL deficiency-induced Rab7 GTPase upregulation is critically involved in lysosomal genesis and distribution in myeloid cells through interacting with the mTOR pathway.

### Rab7 GTPase regulates glucose metabolic switch in myeloid cells

**Figure 4:**
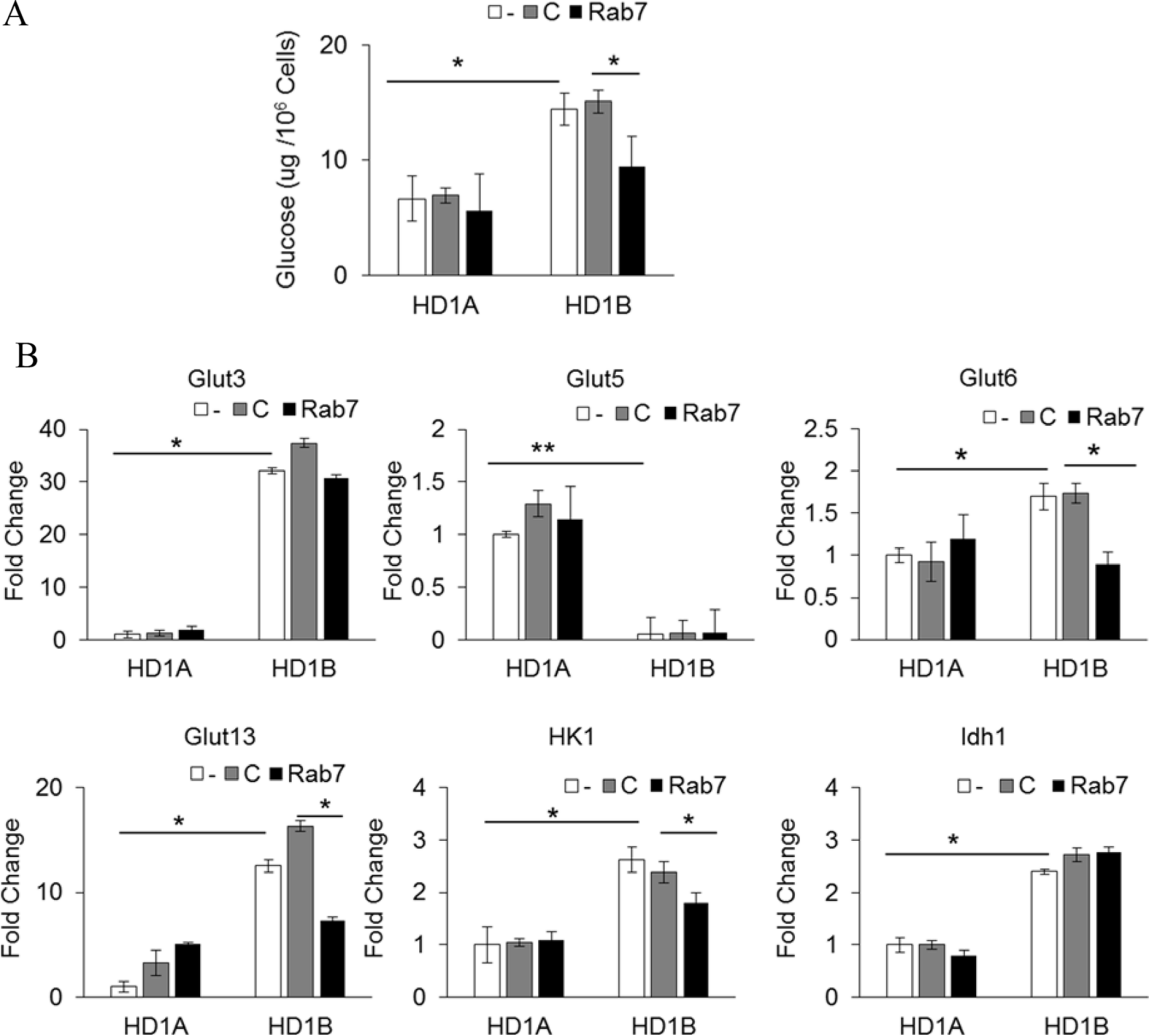
Rab7 GTPase controls glucose metabolism in myeloid cells (**A**) The glucose level was measured in HD1A and HD1B cells with control or Rab7 GTPase siRNA transfection; (**B**) Real time PCR analyses of Glut3, Glut5, Glut6, Glut13, HK1, and IDH1 expression in HD1A and HD1B cells with control or Rab7 GTPase siRNA transfection. The housekeeping gene β*-Actin* was used as internal control. In all above, results are mean ± SD, *n* = 3–4, *p < 0.05. **p < 0.001. -, no transfection; C, transfected with control siRNA; Rab7, transfected with Rab7 siRNA.

As a result of metabolic reprogramming of LAL deficiency and mTOR over-activation, HD1B cells have an increased intracellular glucose level, increased expression of glucose transporter genes and enzymes that are critical for glucose metabolism [7]. As shown in Figure 4A, LAL deficient HD1B cells have a much higher level of the intracellular glucose level than that of wild type HD1A cells. Knocking down Rab7 GTPase significantly reduced the glucose level in HD1B cells. Among four glucose transporter genes (GluT3, Glut5, Glut6 and Glut13) that were changed expression in HD1B cells vs HD1A cells, expression of Glut6 and Glut13 was selectively reduced by Rab7 GTPase knocking down (Figure 4B). This steady-state of mRNA levels could be due to either decreased expression or increased degradation or both. The increased expression of HK1 in HD1B cells was reduced by Rab7 GTPase knocking down while the increased expression of IDH1 in HD1B cells was not (Figure 4B). This concludes that Rab7 GTPase controls the glucose consumption switch in *lal^−/−^* MDSCs and partially overlaps with mTOR over-activation.

### Rab7 GTPase controls ROS production and mitochondrial membrane potential

**Figure 5:**
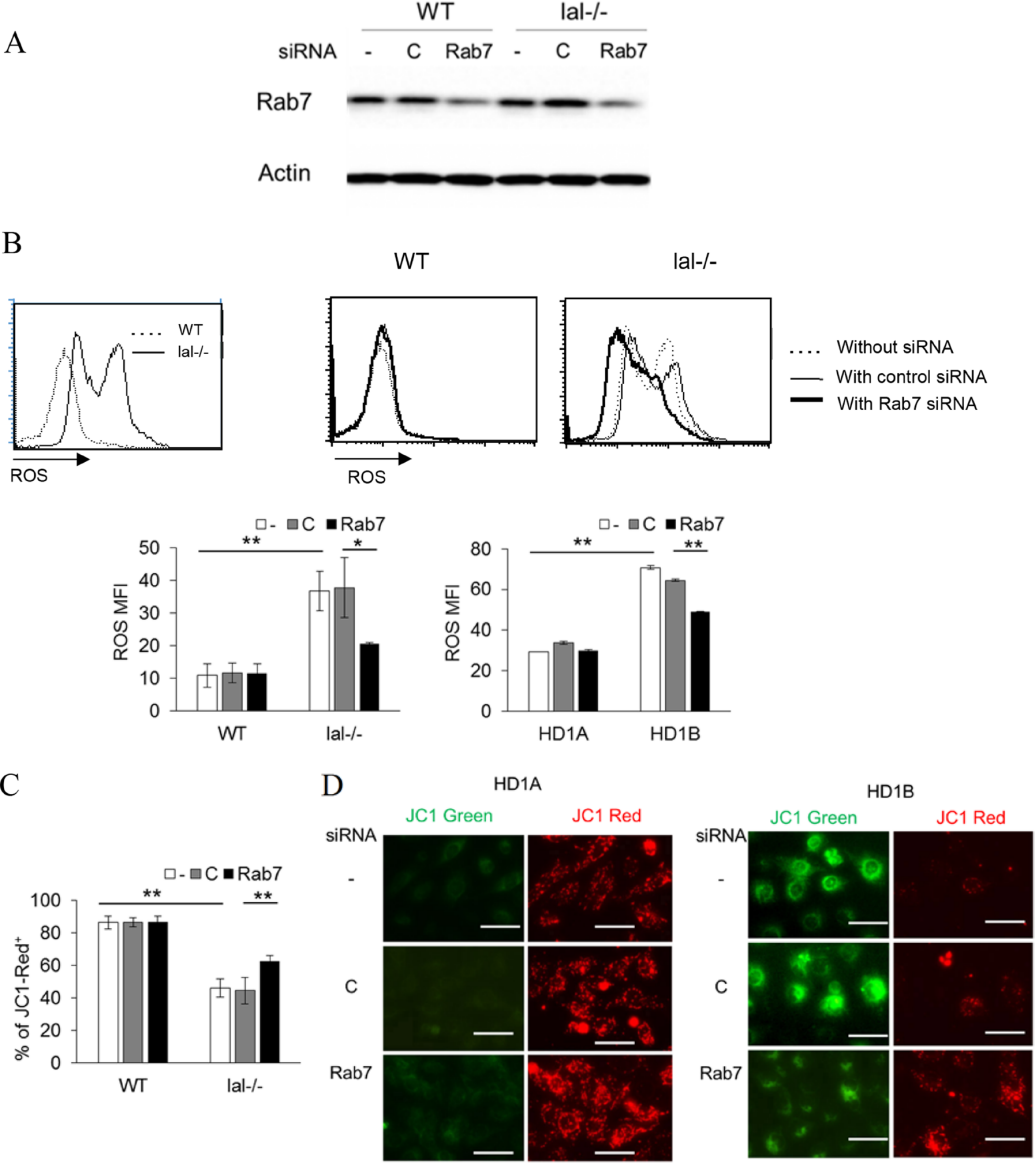
Rab7 GTPase controls ROS production and the mitochondrial membrane potential (**A**) Western blot analysis of Rab7 GTPase expression in wild type and *lal^−/−^* bone marrow Ly6G^+^ cells with control or Rab7 GTPase siRNA transfection for 2 d. Actin was used as loading control. Results are representative of three independent experiments; (**B**) ROS production in wild type and *lal^−/−^* bone marrow Ly6G^+^ cells, or in HD1A and HD1B myeloid cells with control or Rab7 GTPase siRNA transfection. ROS levels were measured by flow cytometry. Results are mean ± SD, *n* = 4, *p < 0.05, **p < 0.001; (**C**) The mitochondrial membrane potential in wild type and *lal^−/−^* bone marrow Ly6G^+^ cells, with control or Rab7 GTPase siRNA transfection. The mitochondrial membrane potential was measured using JC1 staining by flow cytometry. The results are mean from four independent experiments (*n* = 4), *p < 0.05, **p < 0.001; For A-C, -, no transfection; C, transfected with control siRNA; Rab7, transfected with Rab7 siRNA. (**D**) The mitochondrial membrane potential of HD1A and HD1B cells was measured by fluorescence microscope. Results are representative of three independent experiments, scale bar, 25 μm.

Increased glycolysis and over-activation of the mTOR signaling pathway in LAL deficient myeloid cells resulted in the increased ROS production and mitochondrial membrane potential alteration [7, 14]. Transfection of Rab7 GTPase siRNA effectively blocked the Rab7 GTPase expression level compared to that of control siRNA in bone marrow *lal^−/−^* Ly6G^+^ cells (Figure 5A). Knocking down Rab7 GTPase by siRNA significantly reduced the ROS production in *lal^−/−^* Ly6G^+^ cells. This result was further confirmed in MDSCs-like HD1B cells (Figure 5B). The damaged mitochondrial membrane potential was a major contributing factor of ROS over-production. There were more healthy mitochondria (JC-1 red staining cells) in wild type Ly6G^+^ cells and HD1A cells than those in *lal^−/−^* Ly6G^+^ and HD1B cells (Figure 5C–5D). Rab7 GTPase siRNA knocking down partially reversed damaged mitochondria (JC-1 green staining cells) to healthy mitochondria in Ly6G^+^ cells and HD1B cells (Figure 5C–5D).

### Rab7 GTPase controls MDSCs differentiation, trans-EC migration and T cell suppression

**Figure 6:**
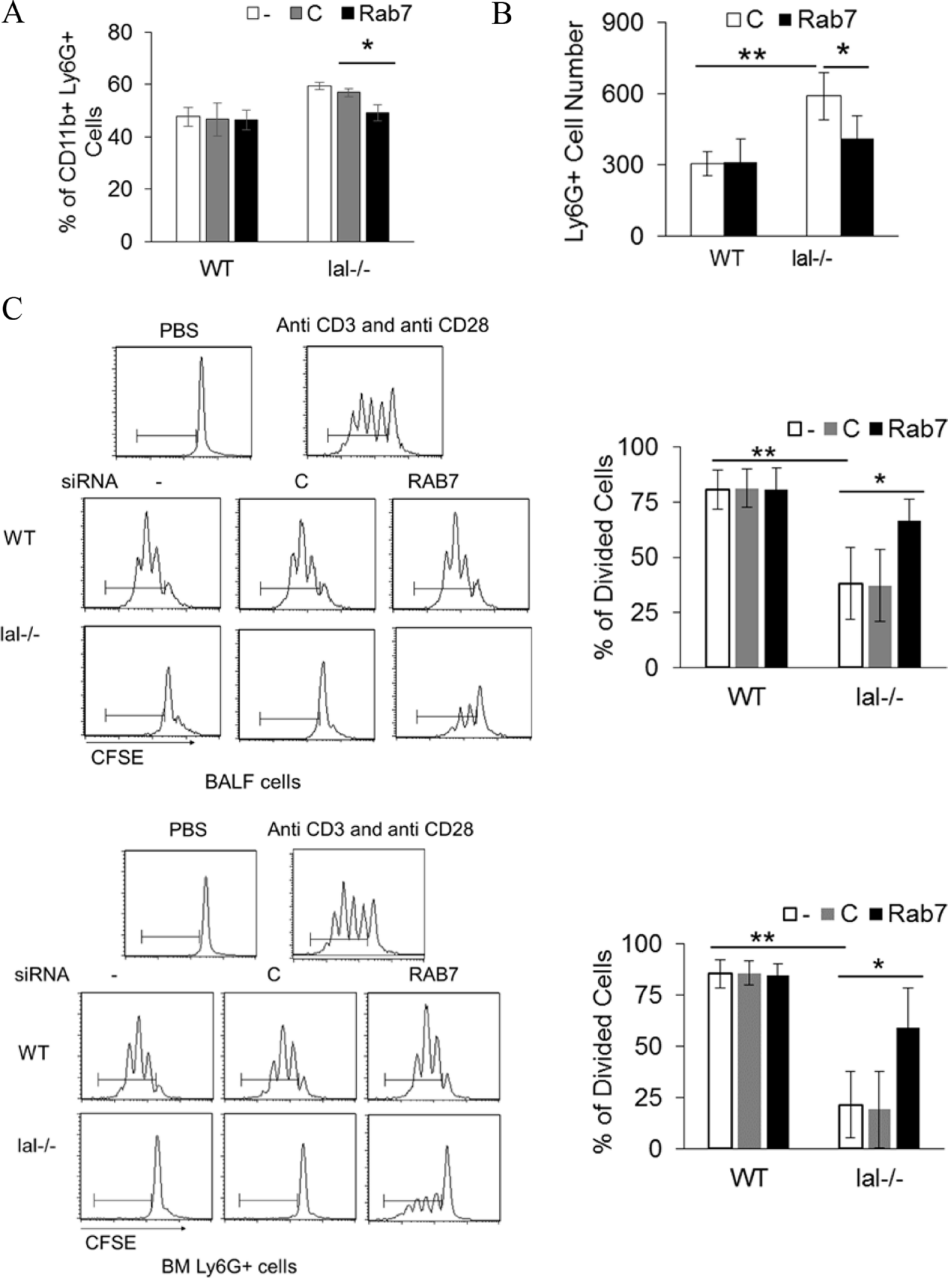
Rab7 GTPase controls MDSCs differentiation and T cell suppression (**A**) Wild type or *lal^−/−^* Lin^−^ cells were isolated from 3-month old mice, and transfected with control or Rab7 GTPase siRNA for 5 d. Differentiated CD11b^+^Ly6G^+^ cells were measured by flow cytometry; (**B**) Transendothelial migration of wild type or *lal^−/−^* bone marrow Ly6G^+^ cells that were transfected with control or Rab7 GTPase siRNA; (**C**) T cell suppression of wild type or *lal^−/−^* Ly6G^+^ cells that were transfected with control or Rab7 GTPase siRNA by flow cytometry. *lal^−/−^* Ly6G^+^ cells were isolated from the bone marrow or BALF. Representative T cell *in vitro* proliferation profiles are shown on the left and the statistical analyses are shown on the right. In all above, results are mean ± SD, *n* = 4, *p < 0.05. **p < 0.001. -, no transfection; C, transfected with control siRNA; Rab7, transfected with Rab7 siRNA.

LAL deficiency results in increased differentiation of MDSCs from lineage negative (Lin^−^) bone marrow cells [22], which is mTOR signaling pathway dependent [14]. To see if Rab7 GTPase participates in MDSCs differentiation, Lin^−^ cells were transfected with control or Rab7 GTPase siRNA. Knocking down Rab7 GTPase reduced CD11b^+^Ly6G^+^ cell differentiation from *lal^−/−^* Lin^−^ cells (Figure 6A). *lal^−/−^* Ly6G^+^ cells have much stronger trans-endothelial ability than that of wild type Ly6G^+^ cells, which is mediated by mTOR over-activation [15]. Rab7 GTPase siRNA knocking down reduced bone marrow derived *lal^−/−^* Ly6G^+^ cells’ trans-endothelial migration (Figure 6B). In organs, *lal^−/−^* MDSCs exhibit very strong suppression on T cells through over-activation of mTOR [14]. Knocking down Rab7 GTPase in bone marrow or bronchoalveolar lavage fluid (BALF) derived *lal^−/−^* Ly6G^+^ cells reduced their suppression of T cell proliferation (Figure 6C).

### Rab7 GTPase controls MDSCs stimulation of tumor cell proliferation, growth and invasion

**Figure 7:**
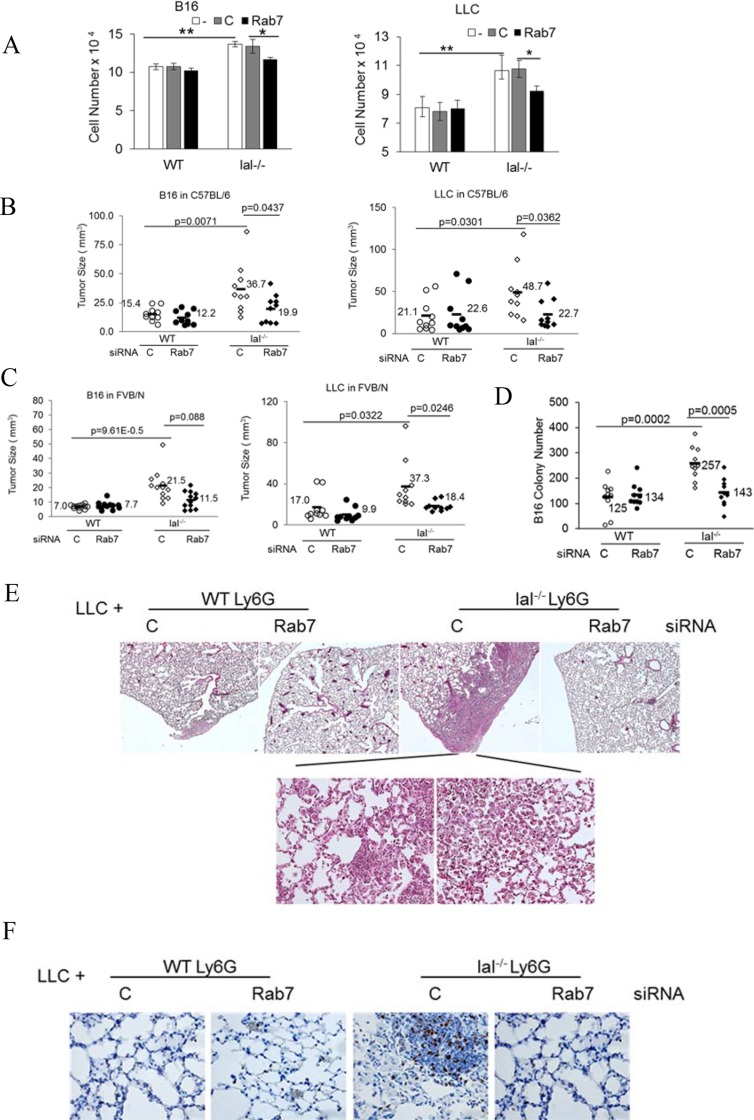
Rab7 GTPase controls tumor stimulation of lal−/− MDSCs (**A**) Ly6G^+^ cells from the wild type or *lal^−/−^* bone marrow were transfected with control or Rab7 GTPase siRNA for 24 h, and 2 × 10^5^ transfected Ly6G^+^ cells were co-cultured with B16 melanoma cells (2 × 10^4^) or LLC cells (2 × 10^4^) *in vitro*. Cells were counted 48 h later. Data were expressed as mean ± SD, *n* = 5. *P < 0.05, **P < 0.001; -, no transfection; C, transfected with control siRNA; Rab7, transfected with Rab7 siRNA. (**B**) Ly6G^+^ cells from the syngeneic C57BL/6 wild type or *lal^−/−^* bone marrow were transfected with control or Rab7 GTPase siRNA for 24 h. Transfected Ly6G^+^ cells were mixed with B16 melanoma cells (2 × 10^5^) or LLC cells (5 × 10^5^) (3:1), followed by subcutaneously flank injection into C57BL/6 wild type recipient mice. Tumor sizes were measured at post injection day 10. The average tumor size of each group and *p* values are marked with *n* = 10; (**C**) Ly6G^+^ cells from the allogeneic FVB/N wild type or *lal^−/−^* bone marrow were transfected with control or Rab7 GTPase siRNA for 24 h. Transfected Ly6G^+^ cells were mixed with B16 melanoma cells (2 × 10^5^) or LLC cells (5 × 10^5^) (3:1), followed by subcutaneously flank injection into the FVB/N wild type recipient mice. Tumor sizes were measured at post injection day 10. The average tumor size of each group and *p* values are marked with *n* = 12; (**D**) Ly6G^+^ cells from C57BL/6 wild type or *lal^−/−^* bone marrow were transfected with control or Rab7 GTPase siRNA for 24 h. Transfected Ly6G^+^ cells (2.5 × 10^6^) were mixed with B16 melanoma cells (5 × 10^5^) (5:1), followed by intravenously injection into C57BL/6 wild type recipient mice through tail vein. B16 melanoma colony numbers were counted at 2 weeks post injection. The average colony number of each group and *p* values are marked with *n* = 10; (**E**) Ly6G^+^ cells from C57BL/6 wild type or *lal^−/−^* bone marrow were transfected with control or Rab7 GTPase siRNA for 24 h. Transfected Ly6G^+^ cells (2.5 × 10^6^) were mixed with LLC cells (5 × 10^5^) (5:1), and intravenously injected into C57BL/6 wild type recipient mice through tail vein. Lung histology of LLC invasion was examined at 2 months post injection. The representative H & E sections were shown, *n* = 10 mice; (**F**) Ki67 staining of E. For A-F: -, no transfection; C, transfected with control siRNA; Rab7, transfected with Rab7 siRNA.

We are the first group showing that MDSCs directly stimulate proliferation of various tumor cells *in vitro*, tumor growth *in vivo* and metastatic invasion, which are mediated by over-activation of mTOR [6]. Rab7 GTPase siRNA transfected bone marrow Ly6G^+^ cells were co-cultured with B16 melanoma cells or LLC cells *in vitro*. Both B16 melanoma cells and LLC cells had significantly increased cell numbers when co-cultured with *lal^−/−^* Ly6G^+^ cells than those with wild type Ly6G^+^ cells. Knocking down Rab7 GTPase reduced ability of *lal^−/−^* Ly6G^+^ cells to stimulate tumor cell proliferation *in vitro* (Figure 7A).

Ly6G^+^ cells from C57BL/6 or FVB/N mice were transfected with control or Rab7 GTPase siRNA, and mixed with B16 melanoma cells or LLC cells for subcutaneous injection in the flank sites of corresponding wild type C57BL/6 or FVB/N recipient mice. At 10 days post injection, the sizes of B16 and LLC tumors grew bigger when co-injected with the *lal^−/−^* Ly6G^+^ cells than those with the wild type Ly6G^+^ cells in both syngeneic C57BL/6 recipient mice (Figure 7B) and allogeneic FVB/N recipient mice (Figure 7C). In both syngeneic and allogenic recipient mice, knocking down Rab7 GTPase by siRNA in wild type Ly6G^+^ cells did not show significant changes in tumor growth. Knocking down Rab7 GTPase in *lal^−/−^* Ly6G^+^cells significantly reduced their tumor growth-stimulating ability in syngeneic C57BL/6 mice for both B16 melanoma and LLC tumor (Figure 7B). In allogeneic FVB/N recipient mice, only LLC tumor growth was affected whereas B16 melanoma had no statistical difference (Figure 7C).

Likewise, knocking down Rab7 GTPase of wild type Ly6G^+^ cells did not show significant changes in tumor invasion of B16 melanoma, but knocking down Rab7 GTPase of *lal^−/−^* Ly6G^+^ cells significantly reduced invasion of B16 melanoma (Figure 7D). A similar observation has been made in the LLC tumor model, in which LLC cell co-injection with *lal^−/−^* Ly6G^+^ cells transfected with control siRNA showed tumor cell invasion in the lung (more Ki67 positive staining), whereas knocking down Rab7 GTPase abolished LLC cell invasion in the lung (Figure 7E and 7F). No LLC invasion was observed when LLC cells were co-injected with wild type Ly6G^+^ cells regardless of control or Rab7 GTPase siRNA transfection.

## DISCUSSION

Fatty acids and glucose are two main sources for fueling energy generation in mitochondria through two oxidation processes, β-oxidation and oxidative phosphorylation (OXPHOS). They generate acetyl-CoA to enter the Kreb cycle and make ATP through passing the respiratory chain and building proton potential on the mitochondrial membrane. The deficiency in LAL leads to fatty acid paucity and blocks ATP generation through β-oxidation in myeloid lineage cells. As a result, myeloid cells undergo a major metabolic shift to overuse the glycolytic pathway in order to compensate energy deficit. Overuse of the glycolytic pathway caused impaired mitochondrial function and increased expression of respiratory chain proteins (including NADH dehydrogenases, cytochrome proteins, ATPases and mitochondrial ribosomal proteins) with penalties: leading to oxidative stress and increased ROS production in *lal^−/−^* MDSCs [7, 10, 14]. High levels of ROS allow for the stimulation of cell proliferation, induction of genetic instability, and evasion from senescence [23].

As we reported previously, the mTOR complexes coordinate metabolic reprogramming in MDSCs [7, 10, 14]. Membrane trafficking causes mTOR to shuttle to lysosomes and regulate mTOR signaling [16, 17]. Therefore, identification of factors that regulate the mTOR trafficking in cells will greatly facilitate understanding of MDSCs homeostasis and functions. Affymetrix GeneChip microarray analysis of bone marrow *lal^−/−^* MDSCs has identified several Rab GTPases that were up-regulated [10]. Among them, Rab7 GTPase is more appealing. It has been well documented that Rab7 GTPase is a late endosome-/lysosome-associated small GTPase, perhaps the only lysosome-associated Rab GTPase protein identified to date [24–27]. Rab7 GTPase participates in multiple regulatory mechanisms in endosomal sorting, biogenesis of lysosome and phagocytosis. These processes are closely related to substrates degradation, antigen presentation, cell signaling, cell survival, microbial pathogen infection, and movement of secretory granules [28, 29]. Consistently, mutations or dysfunctions of Rab7 GTPase result in traffic disorders, which cause various diseases, such as cancer, lipid metabolism disease and neuropathy [18, 25, 30]. More evidence has unraveled that Rab7 small GTPase is involved in cancer formation [19–21]. Rab7 small GTPase is an early-induced melanoma driver whose levels can be upregulated in melanoma as part of a lysosomal-associated signature [19]. It has been demonstrated that troglitazone prevents invasion in prostate cancer cell after extracellular acid pH stimuli, in which Rab7 regulates cell surface-directed lysosome trafficking [31]. Troglitazone is a peroxisome proliferator-activated receptor-γ (PPAR-γ) agonist [32]. LAL downstream derivatives serve as ligands for PPAR-γ [3]. In cancer-stimulating MDSCs, we have recently shown that activation of PPARγ by ligand treatment inhibited *lal*^−/−^ MDSCs stimulation of tumor cell growth and invasion *in vivo*, and tumor cell proliferation and migration *in vitro* through correction of *lal*^−/−^ MDSCs transendothelial migration, and differentiation from bone marrow Lin^−^ cells. The corrective effects of the PPARγ ligand on *lal*^−/−^ MDSCs functions were mediated by regulating the mTOR pathway, and subsequently blocking MDSCs ROS overproduction [5].

Here, Rab7 GTPase knocking down resulted in reduced expression of LAMP1 in MDSCs-like HD1B cells along with down regulation of the mTOR downstream signaling (Figure 1A), indicating that lysosomal anchored Rab7 GTPase controls lysosome genesis by regulating the mTOR signaling. LAL deficient HD1B cells increased lysosomal genesis more around the perinuclear region (Figure 3A–3C). Importantly, the protein-protein physical interaction has been detected for the first time between Rab7 GTPase and mTOR subunit in both HD1A and HD1B cell extract and the purified fusion protein system (Figure 1B–1C and Figure 2). It appeared that Rab7 GTPase interacted with the mTOR through its N-terminal heat repeat domain (Figure 2D and 2E). This was further supported by co-localization of Rab7 GTPase and mTOR on lysosomes (Lamp1 staining) in HD1A and HD1B cells (Figure 3D). Rab7 GTPase knockdown reduced lysosomal genesis and relocated lysosomes from the perinuclear area to spread in cytosol of HD1B cells (Figure 3A–3C). Therefore, LAL-controlled Rab7 GTPase is a critical component in modulation of lysosomal genesis and cellular trafficking in myeloid cells.

At the cellular level, we have reported that over-activation of mTOR, the master metabolic regulator, leads to an increased intracellular glucose consumption and increased gene expression of glucose transporters (Gluts) and enzymes that are critically involved in the glycolytic pathway in HD1B cells [7]. This gave us a reason to speculate that mTOR-associated Rab7 GTPase on lysosome may attribute to this metabolic transition. Indeed, knocking down Rab7 GTPase significantly reduced the glucose consumption level and gene expression of glucose transporters (Glut6 and Glut13) and HK1 in HD1B cells (Figure 4A–4B). As a consequence, the increased glycolysis and over-activation of mTOR resulted in the increased ROS production, which was decreased by blocking Rab7 GTPase (Figure 5B) in *lal^−/−^* MDSCs and MDSCs-like HD1B cells. Furthermore, the damaged mitochondrial membrane potential, a major contributing factor for ROS overproduction, was reversed in *lal^−/−^* MDSCs and HD1B cells by knocking down Rab7 GTPase (Figure 5C). These results indicate that Rab7 GTPase not only physically interacts with the mTOR complex, but also influences mTOR cellular functions in myeloid cells.

One manifestation of LAL deficiency in *lal^−/−^* mice is systemic expansion of tumor-promoting MDSCs. This pathogenic phenotype is mainly contributed by two factors that are controlled by mTOR as we reported before. First, increased myelopoiesis is the major cause for MDSCs expansion in *lal^−/−^* mice [22]. Bone marrow *lal^−/−^* Lin^−^ progenitor cells accelerated into differentiation of *lal^−/−^* MDSCs as a result of mTOR over-activation [14]. Inhibition of mTOR-associated Rab7 GTPase reduced MDSCs differentiation from bone marrow *lal^−/−^* Lin^−^ cells (Figure 6A). Second, *lal^−/−^* MDSCs possess a strong ability to penetrate the endothelial membrane under the control of mTOR [15]. Rab7 GTPase inhibition reduced *lal^−/−^*MDSCs trans-endothelial migration significantly (Figure 6B). Upon exiting the bone marrow and infiltration into distal organs (e.g. lung), the hallmark pathogenic feature of *lal^−/−^* MDSCs is to suppress anti-tumor immunity by down regulating T cell proliferation. This suppressive activity was decreased by Rab7 GTPase knockdown in either bone marrow or BALF derived *lal^−/−^* MDSCs (Figure 6C).

Anti-tumor immunity is an indirect effect by MDSCs to eradicate tumor initiation, progression and invasion in *lal^−/−^* mice, which often takes a longer time to respond. Given the fact that *lal^−/−^* MDSCs and tumor cells are often coexist and traveling together in the body, direct stimulation of tumor cells by *lal^−/−^* MDSCs as we showed recently [6] is a quick and more profound effect on tumor growth and spreading. This direct tumor stimulating effect of *lal^−/−^* MDSCs is also controlled by mTOR. In co-culture and co-injection of *lal^−/−^* MDSCs with tumor cells, Rab7 GTPase knockdown of *lal^−/−^* MDSCs reduced proliferation of B16 melanoma cells and LLC cells *in vitro* (Figure 7A), tumor growth in both syngeneic and allogeneic recipient mice (Figure 7B–7C), and invasion (Figure 7D–7F), suggesting that Rab7 GTPase is critically involved in the LAL/mTOR axis to control tumorigenesis.

In summary, Rab7 GTPase plays a key role in regulating MDSCs development, differentiation, trans-endothelial migration, anti-tumor immunity and direct tumor stimulation through physical interaction with the mTOR complexes. Trafficking regulation of lysosomal anchored proteins in MDSCs such as mTOR and Rab GTPases provides a new direction for immunotherapy in cancer treatment.

## MATERIALS AND METHODS

### Animal care

All scientific protocols involving the use of animals in this study have been approved by the Institution Animal Care and Usage Committee (IACUC) of Indiana University School of Medicine (Indianapolis, IN) and followed the guidelines established by the Panel on Euthanasia of the American Veterinary Medical Association. Protocols involving the use of recombinant DNA or biohazard materials have been approved by the Institutional Biosafety Committee and followed the guidelines established by the National Institutes of Health. Animals were housed under IACUC-approved conditions in a secured animal facility at Indiana University School of Medicine and were regularly screened for common pathogens. Experiments involving animal sacrifice used an approved euthanasia protocol.

### Western blot analysis

Western blot analysis of HD1A and HD1B cells was performed following our previous procedure [7, 8].

### Purification of recombinant Rab7 GTPase and mTOR truncated fusion proteins

Total mRNAs were isolated from HD1B cells and reverse transcribed into cDNAs. The full-length mouse Rab7 GTPase coding region and mTOR fragment coding regions were amplified by PCR from cDNAs and subcloned into the pGEX-4T-1 vector (GE life science, Pittsburgh, PA), which was confirmed by DNA sequencing. Rab7 GTPase-GST fusion protein was expressed in BL21 E. coli by 100 μM IPTG induction and purified by Glutathione Sepharose 4 Fast Flow (GE life science). GST was removed from GST-Rab7 GTPase fusion protein by thrombin cleavage. To get the soluble fusion protein, the inclusion bodies were dissolved in 6M urea and slowly renatured by adding 10 volume buffer (25 mM Tris pH7.5). Centricon (Millipore, Billerica MA) was used to remove additional urea, and the final urea concentration was below 0.6 mM.

### Immuno-precipitation assay

Rabbit IgG or rabbit anti-Rab7 GTPase antibody (9367S, Cell signaling, Beverly, MA) was incubated with 200 μl cell lysate of HD1A or HD1B cells (equivalent to 1–2 × 10^6^ cell) at 4^°^C for 2 h. Protein A agarose (50% bead slurry, 20 μl) was added and incubated for 1 h at 4°C and washed with cell lysis buffer (Cell signaling) 4 times. The beads were boiled in SDS sample buffer before loaded on SDS-PAGE. Proteins associated with the Rab7 GTPase protein were detected by Western blotting analysis using anti-mTOR antibody (2983S, Cell signaling, Beverly, MA).

### Pulldown assay

Glutathione-agarose beads (50% slurry, 40 μl) were incubated with 100 pmole of GST (2.7 μg), or GST-Rab7 GTPase (5.1 μg) in 160 μl wash buffer (50 mM Tris, 150 mM NaCl, pH8.0) in a 0.5 ml micro tube at room temperature for 30 minutes, and washed with buffer for 3 times. The beads were incubated with 200 μl cell lysate of HD1A or HD1B cells (equivalent to 1–2 × 10^6^ cells) at 4^°^C for 2 h and washed four times with 500 μl of wash buffer. The beads were boiled in SDS sample buffer before loading on SDS-PAGE.

To evaluate the interaction between Rab7 GTPase and mTOR truncated fragments, glutathione-agarose beads (50% slurry, 40 μl) were incubated with 100 pmole of GST (2.7 μg), GST-mTOR-1A (7.3 μg), GST-mTOR-1B (7.04 μg), GST-mTOR-M2a2 (5.7 μg), GST-mTOR-M2b (4.8 μg), GST-mTOR-M3 (12.6 μg), or GST-mTOR-M4 (7.4 μg) in 160 μl wash buffer (50 mM Tris, 150 mM NaCl, pH8.0) in 0.5 ml micro tube at room temperature for 30 minutes, and washed with wash buffer for 3 times. The beads were incubated with 200 μl cell lysate of HD1A or HD1B cells (equivalent to 1 – 2 × 10^6^ cells) at 4°C for 2 h and washed four times with 500 μl of wash buffer. The beads were boiled in SDS sample buffer before loading on SDS-PAGE. Proteins associated with the mTOR truncated fragment fusion protein were detected by Western blotting using anti-Rab7 GTPase antibody. Pulldown of recombinant Rab7 GTPase (after GST removal) by various GST-mTOR fragment fusion proteins was performed similarly.

### Glucose measurement

The concentration of glucose in HD1A or HD1B cells was measured by glucose assay kit (Sigma) according the manufacturer’s instruction [7].

### Real time PCR

Total RNAs from HD1A or HD1B cells were purified using the Qiagen total RNA purification kit (Qiagen, Valencia, CA). cDNAs were generated by SurperScript^TM^ III (Invitrogen). Real-time PCR for *Glut1-Glut13, HK1-HK3, IDH1* and the house keeping gene β-Actin were performed on a StepOnePlusReal-Time PCR System (Applied Biosystems) using Power SYBR^®^ Green PCR Master Mix (Applied Biosystems). The (2^−ΔΔCt^) algorithm was used to determine the relative gene expression level [7].

### Rab7 GTPase siRNA sequences

Three sets of Rab7 siRNAs were designed and synthesized by Integrated DNA Technologies using Custom Dicer-Substrate siRNA software.

### Rab7 siRNA1

Sense, 5′ rArCrArGrGrArArArCrArGrArArGrUr GrGrArArCrUrGrUAC

Antisense, 5′ rGrUrArCrArGrUrUrCrCrArCrUrUr CrUrGrUrUrUrCrCrUrGrUrUrU;

### Rab7 siRNA2

Sense, 5′ rGrUrUrGrUrGrUrUrGrGrGrArArAr CrArArGrArUrUrGrACmC

Antisense, 5′ rGrGrUrCrArArUrCrUrUrGrUr UrUrCrCrCrArArCrArCrArArCrArA;

### Rab7 siRNA3

Sense, 5′ rGrGrArArGrArArArGrUrGrUrUr GrCrUrGrArArGrGrUrCAT

Antisense, 5′ rArUrGrArCrCrUrUrCrArGrCrAr ArCrArCrUrUrUrCrUrUrCrCrUrA)

### Reactive oxygen species (ROS) measurement

Freshly isolated total bone marrow cells of wild type or *lal^−/−^* mice were transfected with control or Rab7 GTPase siRNAs for 3 days. The ROS level in CD11b^+^Ly6G^+^ cells was gated and measured by flow cytometry as previously described [10]. The ROS level in HD1A and HD1B cells was measured similarly.

### Mitochondrial membrane potential assay

HD1A and HD1B cells were transfected with control or Rab7 GTPase siRNA in 24-well plate for 3 d. The mitochondrial membrane potential was measure as described before [10]. For Ly6G^+^ bone marrow cells, freshly isolated whole bone marrow cells from wild type or *lal^−/−^* mice were transfected with control or Rab7 GTPase siRNAs for 2 d, stained with the JC-1 (2 μM) and anti-Ly6G APCcy7 antibody (47-5931-82, eBiosciences), and analyzed for PE(red) and FITC(green) fluorescent cells in Ly6G^+^ gated cells by flow cytometry.

### Immunofluorescence staining

HD1A and HD1B cells were fixed for 15 min in 4% paraformaldehyde, and permeabilized for 10 min in 0.02% Triton X 100. After washing, cells were blocked with 5% normal goat serum in 1 x PBS for 1 h followed by incubation of primary rat anti-lysosomal associate membrane protein 1 (LAMP1) antibody (1:500) (sc1992, Santa Cruz, Dallas, TX), mouse anti-mTOR (1:500) (4517S, Cell Signaling), rabbit anti-mTOR (1:500) (2983, Cell Signaling), rabbit anti-Rab7 GTPase (1:500) (9367S, Cell Signaling) overnight. On the second day, cells were incubated with fluorescent conjugated secondary antibody for 1 h. Fluorescent images were examined under the Nikon fluorescence microscope.

### Bone marrow-derived lineage negative (Lin-) cell differentiation *in vitro*

Bone marrow cells were isolated from wild type or *lal^−/−^* mice (8 to 10 weeks of age). A previously described procedure was used to isolate Lin^−^ cells [33]. The cells were transfected with control or Rab7 GTPase siRNAs. Five days after *in vitro* culturing in RPMI1640 plus 10% FCS, CD11b^+^Ly6G^+^ cells derived from Lin^−^ cells were gated and analyzed by flow cytometry.

### Isolation of pulmonary endothelial cells (ECs) and Transwell assay

ECs isolation and transwell assay were performed based on our published protocols [15].

### T cell proliferation *in vitro*

Proliferation of CD4^+^ T cells that were incubated with control or Rab7 GTPase siRNAs transfected bone marrow Ly6G^+^ cells was evaluated as carboxyfluorescein diacetate succinimidyl diester (CFSE, Molecular Probes) dilution peaks by flow cytometry [14].

### Cancer cell growth *in vitro* and *in vivo*

Freshly isolated bone marrow Ly6G^+^ cells from the wild type or *lal^−/−^* mice were transfected with control or Rab7 GTPase siRNAs for 24 h, and 2 × 10^5^ transfected Ly6G^+^ cells were co-cultured with B16 melanoma cells or LLC cells (2 × 10^4^). After 48 h co-culture, unattached Ly6G^+^ cells were removed by washing, and the number of attached B16 melanoma cells or LLC cells was counted.

Freshly isolated Ly6G^+^ cells from the C57BL/6 or FVB/N wild type or *lal^−/−^* bone marrow were transfected with control or Rab7 GTPase siRNAs for 24 h, and 6 × 10^5^ of transfected Ly6G^+^ cells were mixed with 2 × 10^5^ B16 melanoma cells (3:1), or 15 × 10^5^ of transfected Ly6G^+^ cells were mixed with 5 × 10^5^ LLC cells (3:1) and incubated for 1 h, which were injected subcutaneously at left and right flank sites of wild type C57BL/6 or FVB/N recipient mice, respectively. The tumor size was measured 10 d post-injection with calipers. The tumor volumes were determined using the formula: (length x width^2^) /2 [6]. At the end of the experiment, the animals were euthanized.

### Cancer cell invasion

Freshly isolated bone marrow Ly6G^+^ cells from C57BL/6 wild type or *lal^−/−^* mice were transfected with control or Rab7 GTPase siRNA for 24 h. Transfected Ly6G^+^ cells (2.5 × 10^6^) were mixed with B16 melanoma cells (5 × 10^5^) (5:1) and incubated for 1 h, which were intravenously injected into C57BL/6 wild type recipient mice through tail vein. For the B16 melanoma invasion, B16 melanoma colony numbers were counted at 2 weeks post injection. For LLC invasion, lung histology and Ki67 immunohistochemical staining were examined at 2 months post injection.

### Statistical analysis

A paired Student’s *t-test* or ANOVA was used to evaluate the significance of the differences. The results were mean ± SD of at least three independent experiments.

## SUPPLEMENTARY MATERIALS FIGURES



## Abbreviations

BALF: bronchoalveolar lavage fluid
CFSE: carboxyfluorescein diacetate succinimidyl diester
ECs: endothelial cells
Gluts: glucose transporters
LAL: lysosomal acid lipase
*lal*^−/−^: LAL deficient
LAMP1: lysosomal associate membrane protein 1
Lin^−^: lineage negative
MDSCs: myeloid-derived suppressor cells
mTOR: mammalian target of rapamycin
OXPHOS: oxidative phosphorylation
PI3K: phosphoinositide 3-kinase
ROS: reactive oxygen species
