# Supplementary material for: Rab7 GTPase controls lipid metabolic signaling in myeloid-derived suppressor cells
Source: Oncotarget. 2017 Mar 16;8(18):30123–37. doi: 10.18632/oncotarget.16280 (PMC5444731; doi:10.18632/oncotarget.16280)
Supplement: Supplementary file 1 [file oncotarget-08-30123-s001.pdf]

Rab7 GTPase controls lipid metabolic signaling in myeloid-derived suppressor cells

Supplementary Materials

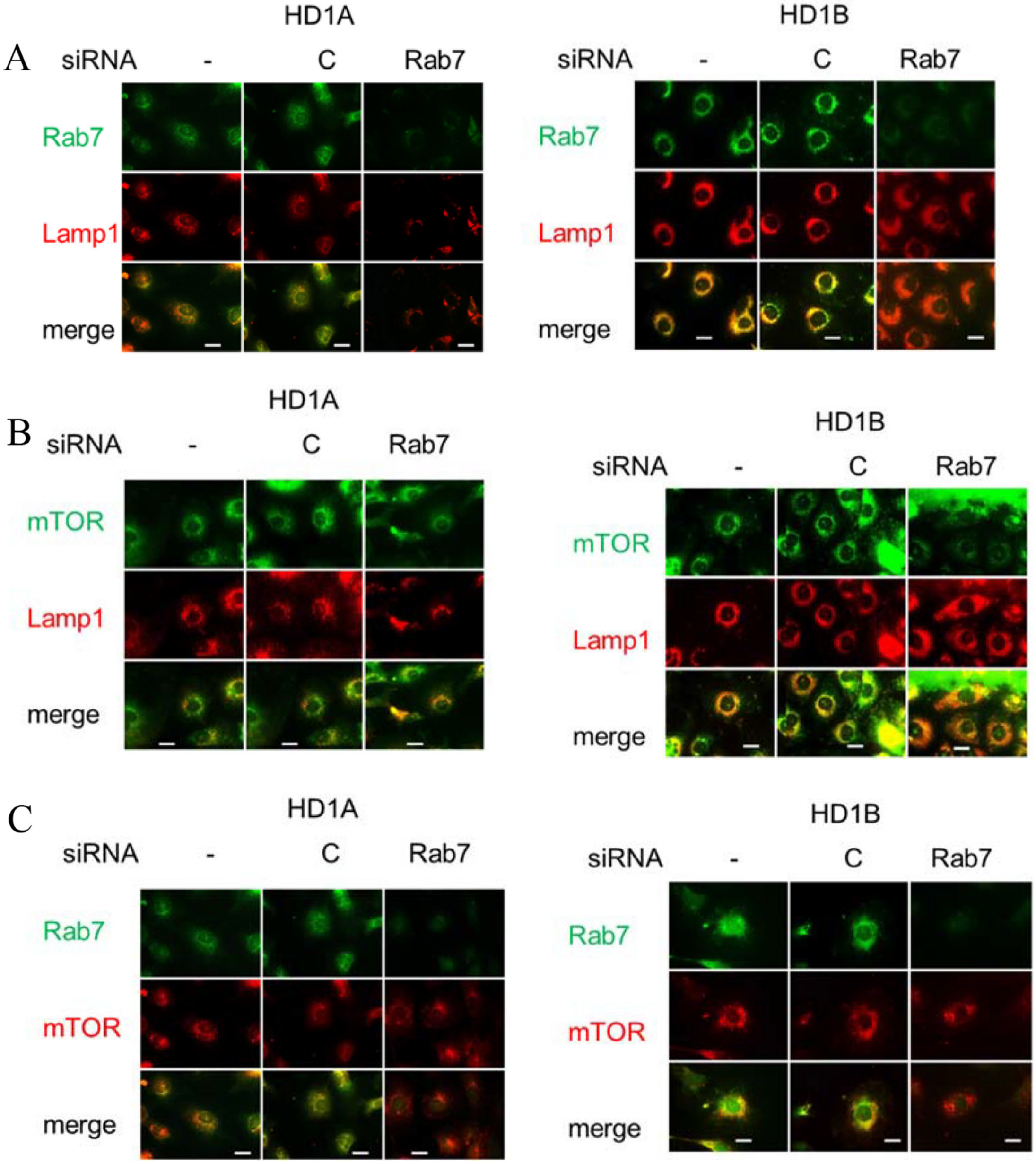

Supplementary Figure 1: Rab7 GTPase controls lysosome genesis. (**A**) Co-localization of Rab7 GTPase and LAMP-1 by immunofluorescent staining in HD1A and HD1B cells with control or Rab7 GTPase siRNA treatment for 3 days; (**B**) Co-localization of mTOR and LAMP-1 by immunofluorescent staining in HD1A and HD1B cells with control or Rab7 GTPase siRNA treatment for 3 days. The mTOR staining intensity was reduced by Rab7 GTPase siRNA knocking down; (**C**) Colocalization of Rab7 GTPase and mTOR by immunofluorescent staining in HD1A and HD1B cells with control or Rab7 GTPase siRNA transfection for 3 days. The mTOR staining intensity was reduced by Rab7 GTPase siRNA knocking down. From A-C, results are representative of five independent experiments, scale bar, 20 µm. -, no transfection; C, transfected with control siRNA; Rab7, transfected with Rab7 siRNA.
